# Body Size and Body Weight in *Apis cerana*: Associations with Geographic, Climatic, and Productive Traits for Bee Breeding

**DOI:** 10.3390/life16060980

**Published:** 2026-06-10

**Authors:** Hanbing Lu, Xinru Zhang, Bangrong Wei, Guoling Wang, Xinyi You, Xinying Qu, Lingjun Xin, Xiao Chen

**Affiliations:** 1 State Key Laboratory of Resource Insects, Institute of Apicultural Research, Chinese Academy of Agricultural Sciences, Beijing 100193, China; 821012530463@caas.cn (H.L.); 82101235489@caas.cn (X.Q.); 2 College of Bioscience and Resource Environment, Beijing University of Agriculture, Beijing 102206, China; 202330112016@bua.edu.cn (X.Z.); xinlingjun@bua.edu.cn (L.X.); 3 Chongqing Nanchuan District Animal Husbandry, Veterinary and Fishery Center, Chongqing 408499, China; 19930688709@126.com (B.W.); 19930690389@126.com (G.W.); 13950603110@163.com (X.Y.)

**Keywords:** colony performance, environmental adaptation, genetic improvement, honey bee, morphometric indicator

## Abstract

*Apis cerana* (*A. cerana*) is a native and widely managed honey bee species in China. Body size and body weight are crucial breeding traits, as colonies possessing individuals with large body weight tend to be healthier and exhibit high productivity. This study aimed to clarify the relationships between body size and body weight in *A. cerana* and to evaluate their associations with geographic, climatic, and colony productive traits for selective breeding. Body size and body weight were measured in virgin queens, drones, and workers from Jinfo Mountain, Chongqing, and additional measurements of queens and drones were implemented in five other regions across China. Linear mixed-effects models confirmed that body size had a significant positive effect on body weight in virgin queens, drones, and workers. However, correlations of body-size and body-weight traits among different bee groups were weak and non-significant after FDR correction, indicating that drones or workers cannot be used as direct substitutes for queen body-size traits in the present dataset. Standardized model estimates showed that queen and drone body-size and body-weight traits were consistently negatively associated with annual minimum and annual mean temperatures, but positively associated with latitude after FDR adjustment. Annual precipitation was also negatively associated with queens’ body size, queens’ body weight, and drones’ body size, whereas annual maximum temperature, longitude, and elevation showed no significant associations after FDR adjustment. Moreover, queens’ body size and body weight were significantly positively associated with honey yield, honey yield during the main nectar flow, and colony gentleness after FDR correction, whereas their associations with the number of effective eggs laid by queens, colony strength, and robbery were not significant after FDR correction. These findings suggest that queen body-type traits may serve as useful auxiliary indicators for selecting colonies with higher honey production and gentler behavior, but their relationships with other colony traits should be interpreted cautiously. This research is beneficial for initiating a body size-weight selective breeding program for *A. cerana*, as it can help optimize breeding objectives and accelerate genetic progress.

## 1. Introduction

Honey bees are important contributors to crop pollination and have significant economic value, contributing to plant reproduction, biodiversity maintenance, and agricultural production [1,2]. Bee pollination services contribute billions of dollars annually to agricultural economies [3,4]. Animal pollination, including bee pollination, contributes to the production of many leading global food crop types and improves the yield and quality of many fruits, vegetables, and nuts, thereby supporting global food security [5,6]. Also, products from apiculture, such as honey and royal jelly are health products for humans and help improve physical health. Thus, honey bees possess critical ecological and economic value [7].

Through long-term selective breeding, honey bees have developed characteristics, such as strong adaptability, disease resistance and ease of management [8,9]. Reports on honey bees began to increase in the 1950s, mostly focusing on breeding management and hybrid improvement [10,11]. However, there have been few reports on the correlation analysis and regression equations between body size and body weight of honey bees. In particular, systematic information on the quantitative relationship between body size and body weight in *A. cerana* remains limited, and it is still unclear whether these body-type traits are associated with geographic, climatic, and colony productive traits. This knowledge gap limits the use of body size and body weight as practical selection indicators in *A. cerana* breeding programs. Therefore, it is necessary to clarify the relationships between body size and body weight and to evaluate their potential value for selective breeding. Body size and body weight are important traits in honey bee breeding programs, as they reflect the morphological structure and developmental capacity of honey bees [12]. In apicultural production, beekeepers prefer to select colonies with large body size and high body weight as breeders. Workers with high body weight extend longevity, immune competence and an enhanced ability to cope with pesticides [13]. For queens, it is found that the body weight affects their reproductive ability [14,15]. Body size and body weight are important morphological indicators for evaluating the reproductive quality of queens and drones. In queens, emergence weight and ovariole number are closely associated with reproductive potential and are commonly used as indicators of queen quality [15,16,17]. In drones, body size and body weight are related to sexual maturation, sperm quality, flight performance, and mating success [18,19,20,21,22,23,24]. Therefore, body size and body weight are important traits for assessing reproductive performance in honey bees and may provide useful indicators for colony breeding and selection.

Based on these knowledge gaps, this study aimed to determine whether body size and body weight can serve as practical morphological indicators for selective breeding in *A. cerana*. We hypothesized that body size and body weight are positively associated within queens, drones, and workers; that body traits may show group-specific or cross- group associations; and that queen and drone body traits are associated with geographic, climatic, and colony productive traits. To test these hypotheses, we addressed the following research questions: (1) What is the quantitative relationship between body size and body weight in queens, drones, and workers? (2) Do queen, drone, and worker body traits show significant cross-group associations? (3) How are queen and drone body traits associated with geographic and climatic factors across different regions? (4) Are queen body size and body weight associated with colony production performance and biological traits? By answering these questions, this study provides a data foundation for body size–weight-based selective breeding and for optimizing breeding objectives in *A. cerana*.

## 2. Materials and Methods

### 2.1. Study Area and Sample Collection

The study was conducted in one primary apiary and five additional geographical regions across China. The primary apiary was located in Jinfo Mountain, Chongqing, China, and was used for the within-group body size–body weight analyses of newly emerged virgin queens, drones, and workers. To further evaluate the associations of queen and drone body-type traits with environmental, climatic, and colony productive traits, five additional regions were included: Changbai Mountain, Jilin; Batang, Sichuan; Aba, Sichuan; Jiaoling, Guangdong; and Danzhou, Hainan. These regions represent different geographical and climatic backgrounds and were used for the multi-region analyses of queens and drones. Sample collection and trait measurements in Jinfo Mountain and the five additional regions were conducted during the same seasonal window, from May to July 2025, corresponding to the active breeding and colony development season of *A. cerana* in the study regions.

**Figure 1:**
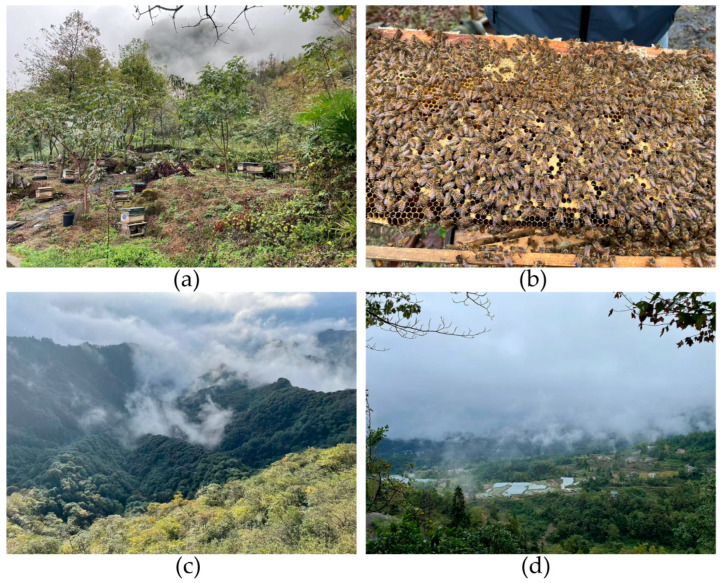
Apiaries, colonies and ecological environment in Jinfo Mountain, Chongqing, China. (**a**) The apiary of *A. cerana* in Jinfo Mountain. (**b**) The colony of *A. cerana* in Jinfo Mountain. (**c**,**d**) The ecological environment of *A. cerana* in Jinfo Mountain.

To investigate the correlations between body size and body weight in *A. cerana*, newly emerged virgin queens, drones, and workers were collected from an apiary in Jinfo Mountain, Chongqing, China (Figure 1). A total of 15 colonies were used to rear virgin queens, drones and workers. For each colony, 10 virgin queens, 10 to 20 drones and 30 to 40 workers were measured. To obtain newly emerged queens, the date of queen-cell formation or capping was recorded during queen rearing. The expected emergence time was estimated based on the developmental schedule of *A. cerana* queens, in which queen development from egg laying to adult emergence takes approximately 16 days, with queen-cell capping occurring at approximately day 8 and adult emergence occurring at approximately day 16 [25]. Accordingly, queen cells at approximately day 15 after egg laying, corresponding to about one day before expected emergence, were placed into a constant temperature incubator (HWS-100, Ningbo Saifu Experimental Instrument Co., Ltd., Ningbo, Zhejiang, China) (34 °C, relative humidity 75% ± 5%, and dark conditions) until the emergence of queens. For workers and drones, the brood combs about to emerge were placed into the same constant temperature incubator (34 °C, relative humidity 75% ± 5%, and dark conditions) until bees’ emergence. After emergence, individual honey bees were placed into 50 mL centrifuge tubes, the colony number was recorded, and each individual was assigned a unique identification number. Body size and body weight were measured within two hours after emergence.

**Figure 2:**
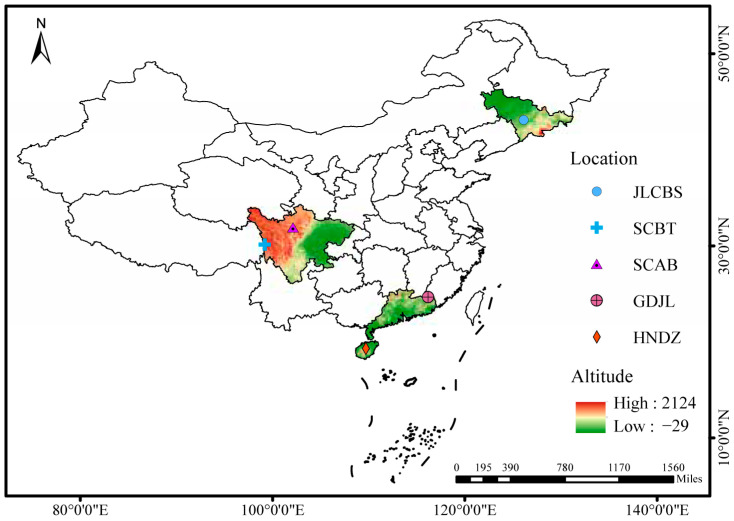
Locations of apiaries for measuring body size and body weight of *A. cerana* across China. JLCBS, Changbai Mountain, Jilin, China; SCBT, Batang, Sichuan, China; SCAB, Aba, Sichuan, China; GDJL, Jiaoling, Guangdong, China; HNDZ, Danzhou, Hainan, China.

To analyze the correlations between body size/weight of queens/drones and environmental and climatic factors, body size and body weight of queens and drones in five other regions of China were measured using the same methods (Figure 2). In each region, 15 colonies were sampled, and 15 virgin queens and 15 drones were measured from each colony. These five-region data were used for the environmental and production-trait analyses, whereas the Jinfo Mountain dataset was used for the within-group body size–weight analyses of virgin queens, drones, and workers.

### 2.2. Body Size and Body Weight Measurements

**Figure 3:**
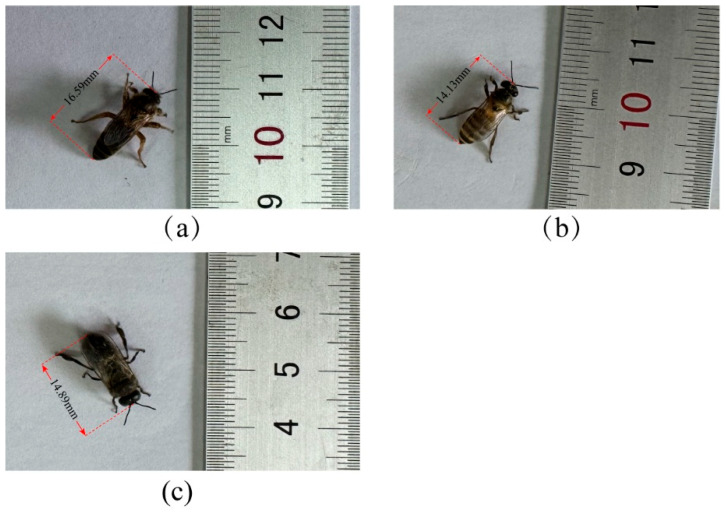
Measurements of the body size of the virgin queen (**a**), the worker (**b**) and the drone (**c**).

When measuring the emergence body weight, the honey bee was placed into a tube and the body weight was recorded using an electronic scale (FA2004, LICHEN, Ningbo Yinzhou Huafeng Electronic Instrument Factory, Ningbo, Zhejiang, China). When measuring the body size, the honey bee was placed on a flat surface, and a scale was placed next to it. Body size measurements were conducted on newly emerged bees approximately within two hours after emergence. At this stage, bees were generally inactive and showed limited movement. To minimize posture-related measurement error, each bee was placed on a flat surface and photographed only when the body was naturally extended along the longitudinal axis. Images in which the bee showed obvious abdominal curvature, curling, or incomplete extension were excluded, and the individual was photographed again until a fully extended posture was obtained. The Leica DMS300 digital microscope system equipped with a CCD camera (Leica Microsystems GmbH, Wetzlar, Germany) was fixed to take a picture of the honey bee, ensuring that the honey bees and the scale were within the same field of view. Following image acquisition, the actual size of honey bees was measured using Adobe Photoshop 2023 (Adobe Inc., San Jose, CA, USA). During measurement, body size was determined along the longitudinal axis of the bee as the straight-line distance from the anterior margin of the head (clypeal anterior edge) to the posterior margin of the terminal abdominal sternite (excluding the sting or caudal appendages) (Figure 3). This whole-body length measurement was selected because it provides a practical and non-lethal estimate of body size for live or newly emerged bees, which is particularly important in large-scale breeding work and queen evaluation. In classical honey bee morphometrics [26], the longitudinal lengths of tergite 3 and tergite 4 are commonly used as size-related abdominal traits. Therefore, the body size measured in a fully extended state was expected to partly reflect variation in abdominal longitudinal dimensions, while also allowing rapid and standardized comparison among queens, workers, and drones. The obtained data of body size and body weight were subsequently subjected to correlation analyses.

### 2.3. Environmental and Climatic Data

To investigate the effects of environmental and climatic factors on body size and body weight, we obtained data of annual maximum temperature, annual minimum temperature, annual mean temperature, and annual precipitation from the National Meteorological Information Center of the China Meteorological Administration (https://data.cma.cn, accessed on 12 January 2026). These temperature and precipitation variables were annual climatic summary variables for each study region. Geographic environmental data, including latitude, longitude, and elevation of the study regions, were obtained from the National Earth System Science Data Center (https://www.geodata.cn, accessed on 13 January 2026) and the Resource and Environment Science and Data Center of the Chinese Academy of Sciences (https://www.resdc.cn, accessed on 13 January 2026).

### 2.4. Measurement of Colony Production and Biological Traits

To investigate whether queens’ body size and body weight were associated with colony production performance and biological traits, we tested the following traits: honey yield (HY, kg/colony/year), honey yield during the main nectar flow (HYMNF, kg/colony), number of effective eggs laid by queen (NEELQ), colony strength (CST, count by combs), robbery and gentleness. Measurements followed the Technical specification for honey bee genetic resource survey (GB/T 45550-2025) and Technical specification for performance testing of honey bees (GB/T 45551-2025) [27,28]. Specifically, HY was the total marketable honey produced per colony per year. HYMNF was the harvestable honey obtained during peak nectar secretion. NEELQ was the mean number of eggs per day that developed into pupae. CST was the number of frames covered by adult workers and brood. Robbery and gentleness were evaluated under standardized conditions and scored as 1, 2, and 3 for weak, moderate, or strong.

### 2.5. Data Analysis

All statistical analyses were performed in R software version 4.6.0 [29]. Spearman’s rank correlation analyses were performed using the stats package. Linear mixed-effects models were fitted using the lme4 package version 2.0-1 [30], and *p*-values for fixed effects were obtained using the lmerTest package version 3.2-1 [31]. Variables were standardized before model fitting using the scale function in R. Multiple testing correction was performed using the Benjamini–Hochberg false discovery rate method with the p.adjust function. Heatmaps and correlation plots were generated using the ggplot2 package version 4.0.3 [32]. Descriptive statistics for body size and body weight are presented as mean ± standard deviation (SD), unless otherwise specified. Fixed-effect estimates from linear mixed-effects models are presented as estimate ± standard error (SE).

To investigate the relationship between body size and body weight within each bee group of *A. cerana* from Jinfo Mountain, Spearman’s rank correlation analysis was first used as a descriptive analysis because body weight data did not fully meet the assumption of normality. To account for the hierarchical sampling design, in which individual bees were sampled within colonies, linear mixed-effects models were then fitted separately for virgin queens, drones, and workers. In each model, body weight was used as the response variable, body size was included as a fixed effect, and colony identity was included as a random intercept. The model structure was as follows: body weight ~ body size + (1|colony). Body size was expressed as mm × 100 to maintain consistency with the graphical presentation. The fixed-effect estimate, standard error, 95% confidence interval, and *p*-value were reported. Spearman’s correlation coefficients were retained for descriptive visualization, whereas the mixed-effects models were used as the primary statistical inference.

To evaluate the associations between queen and drone body-type traits and environmental/climatic factors, each body trait, including queens’ body size, queens’ body weight, drones’ body size, and drones’ body weight, was analyzed separately against each environmental variable. For these analyses, linear mixed-effects models were fitted using each body trait as the response variable and each environmental or climatic factor as the fixed-effect explanatory variable, with colony identity nested within region identity included as random intercepts. The model structure was as follows: body trait ~ environmental variable + (1|Region_ID/Colony_ID). Body traits and environmental variables were standardized before model fitting, and the resulting standardized estimates were used to compare the direction and magnitude of associations among variables. *p*-values from multiple trait–environment tests were adjusted using the Benjamini–Hochberg false discovery rate method. The results were visualized as a heatmap, in which circle size and color represent the magnitude and direction of the standardized estimate, and asterisks indicate significant associations after FDR correction (* FDR-adjusted *p* < 0.05). For the production and biological-trait analysis, queen body size and queen body weight were separately analyzed against HY, HYMNF, NEELQ, CST, robbery, and gentleness. For these analyses, linear mixed-effects models were fitted using each production or biological trait as the response variable and queen body size or queen body weight as the fixed-effect explanatory variable, with colony identity nested within region identity included as random intercepts. The model structure was as follows: production or biological trait ~ QBS or QBW + (1|Region_ID/Colony_ID). Variables were standardized before analysis, and standardized estimates were used to visualize the direction and magnitude of associations. *p*-values from multiple tests were adjusted using the Benjamini–Hochberg false discovery rate method, and only associations with FDR-adjusted *p* < 0.05 were considered statistically significant. To provide a quantitative example of the practical breeding implications, regional mean values were calculated for queen body size and honey-yield traits across the five regions. Ordinary least-squares regressions were then fitted with annual honey yield and honey yield during the main nectar flow as the response variable and queen body size as the explanatory variable. The regression slope was interpreted as the expected change in honey yield per 1 mm increase in queen body size.

## 3. Results

### 3.1. Body Size and Body Weight of A. cerana from Jinfo Mountain, Chongqing

**Figure 4:**
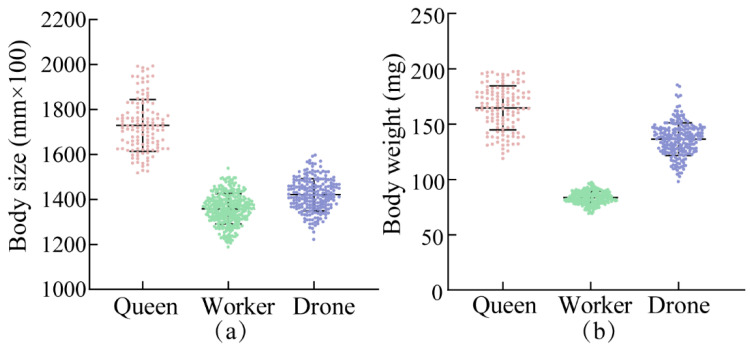
Body size (**a**) and body weight (**b**) of virgin queens, workers, and drones of *A. cerana* in Jinfo Mountain, Chongqing.

As shown in Figure 4, descriptive values are presented as mean ± SD. The emergence body weight of virgin queens was 164.70 ± 19.93 mg, and the body size was 17.29 ± 1.28 mm. The emergence body weight of workers was 83.64 ± 5.69 mg, and the body size was 13.59 ± 0.67 mm. The emergence body weight of drones was 136.45 ± 14.73 mg, and the body size was 14.21 ± 0.71 mm. The coefficients of variation for body size in virgin queens, workers, and drones were low, indicating that body size is relatively stable in *A. cerana*. In contrast, the coefficients of variation for initial body weight in queens and drones exceeded 10%, suggesting substantial variation in body weight. These findings indicate that initial body weight in *A. cerana* has considerable potential for selective breeding.

### 3.2. Relationship Between Body Size and Body Weight Within the Same Bee Group

**Figure 5:**
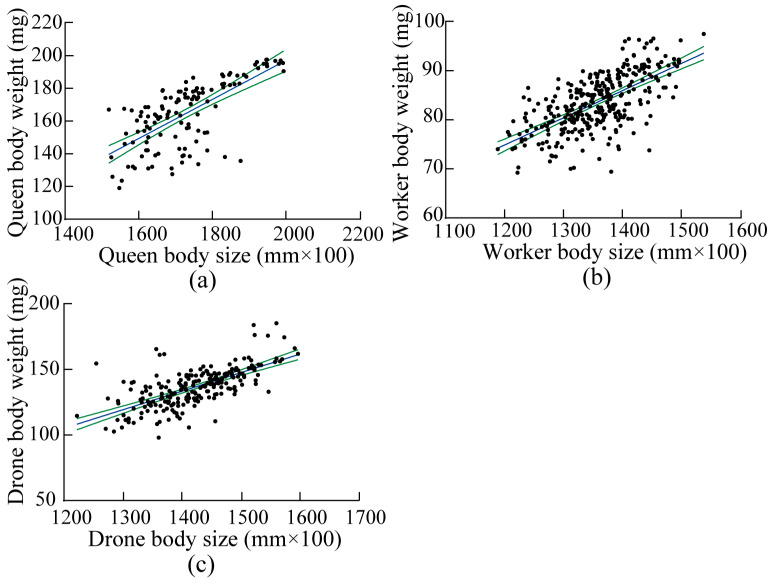
Descriptive correlation plots between body size and body weight in virgin queens (**a**), workers (**b**), and drones (**c**). The fitted lines and confidence intervals are shown only to visualize the overall trends. Spearman’s correlation analysis was used only for graphical presentation. Formal statistical inference was performed using linear mixed-effects models with colony identity included as a random intercept.

Descriptive correlation plots showed positive associations between body size and body weight within each bee group of *A. cerana* from Jinfo Mountain, Chongqing (Figure 5). Because individuals were sampled from the same colonies, linear mixed-effects models were further used to account for colony-level non-independence (Table 1). The mixed-effects models confirmed that body size had a significant positive effect on body weight in virgin queens, drones, and workers. In virgin queens, the fixed-effect estimate of body size was 0.1157 mg per unit of body size (SE = 0.0099; 95% CI: 0.0963–0.1351; *p* < 0.001). In drones, the estimate was 0.0866 mg per unit of body size (SE = 0.0146; 95% CI: 0.0580–0.1151; *p* < 0.001). In workers, the estimate was 0.0337 mg per unit of body size (SE = 0.0043; 95% CI: 0.0253–0.0420; *p* < 0.001). These results indicate that the positive relationship between body size and body weight remained significant after accounting for colony-level random effects.

### 3.3. Correlation of Body Size and Body Weight Among Different Bee Groups

**Figure 6:**
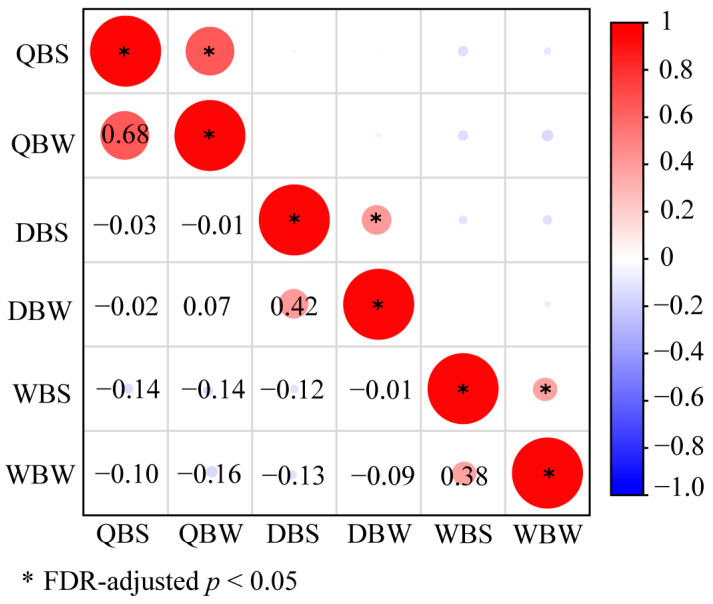
Pairwise correlations of body size and body weight among queens, drones, and workers. QBS, queens’ body size; QBW, queens’ body weight; DBS, drones’ body size; DBW, drones’ body weight; WBS, workers’ body size; WBW, workers’ body weight. Circle size and color represent the magnitude and direction of the correlation coefficient. Values in the lower triangle indicate correlation coefficients. Asterisks indicate significant correlations after FDR correction (* FDR-adjusted *p* < 0.05).

Pairwise correlation analysis was used to visualize the relationships between body size and body weight among queens, drones, and workers (Figure 6). Significant positive correlations were observed between body size and body weight within the same bee group. Specifically, queen body size was positively correlated with queen body weight (QBS–QBW, r = 0.68), drone body size was positively correlated with drone body weight (DBS–DBW, r = 0.42), and worker body size was positively correlated with worker body weight (WBS–WBW, r = 0.38), and these relationships remained significant after FDR correction.

In contrast, the correlations between different bee groups were weak and did not remain significant after FDR correction. For example, the correlations between queen body size and drone body size, queen body size and drone body weight, queen body weight and drone body size, and queen body weight and drone body weight were very low, with coefficients ranging from −0.03 to 0.07. Similarly, correlations between queen traits and worker traits, as well as between drone traits and worker traits, were also weak and non-significant.

These results indicate that body size and body weight are positively associated within each bee group, whereas body-size and body-weight variation is not clearly synchronized among queens, drones, and workers. Therefore, the present results do not support the use of drone or worker body-size traits as direct substitutes for queen body-size traits.

### 3.4. Associations Between Environmental/Climatic Factors and Body-Type Traits

**Figure 7:**
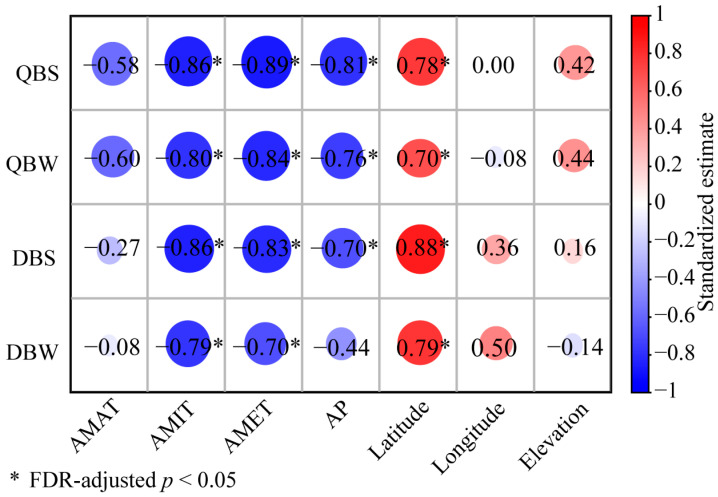
Standardized model estimates for associations between queen and drone body-type traits and environmental/climatic factors. QBS, queens’ body size; QBW, queens’ body weight; DBS, drones’ body size; DBW, drones’ body weight; AMAT, annual maximum temperature; AMIT, annual minimum temperature; AMET, annual mean temperature; AP, annual precipitation. Circle size and color indicate the magnitude and direction of standardized estimates. Asterisks indicate significant associations after FDR correction (* FDR-adjusted *p* < 0.05).

Queens and drones are reproductive bee groups and were therefore included in the environmental analysis. Standardized model estimates revealed clear associations between queen and drone body-type traits and environmental/climatic factors (Figure 7). The strongest negative associations were observed for annual minimum temperature and annual mean temperature. After FDR correction, annual minimum temperature and annual mean temperature were significantly negatively associated with all four body traits, including queens’ body size, queens’ body weight, drones’ body size, and drones’ body weight. Specifically, the standardized estimates for annual minimum temperature and annual mean temperature were −0.86 and −0.89 for queens’ body size, respectively (both FDR-adjusted *p* < 0.05), −0.80 and −0.84 for queens’ body weight, respectively (both FDR-adjusted *p* < 0.05), −0.86 and −0.83 for drones’ body size, respectively (both FDR-adjusted *p* < 0.05), and −0.79 and −0.70 for drones’ body weight, respectively (both FDR-adjusted *p* < 0.05).

Annual precipitation also showed significant negative associations with queens’ body size, queens’ body weight, and drones’ body size, with standardized estimates of −0.81, −0.76, and −0.70, respectively (all FDR-adjusted *p* < 0.05), whereas its association with drones’ body weight was weaker and not significant after FDR correction. In contrast, latitude was significantly positively associated with all four body traits, with standardized estimates of 0.78 for queens’ body size, 0.70 for queens’ body weight, 0.88 for drones’ body size, and 0.79 for drones’ body weight (all FDR-adjusted *p* < 0.05). Annual maximum temperature, longitude, and elevation showed no significant associations with queen or drone body-type traits after FDR adjustment. Overall, these results indicate that queen and drone body size and body weight tended to increase with latitude but decrease with higher annual minimum and mean temperatures, while annual precipitation mainly showed negative associations with queen traits and drone body size in the present dataset.

### 3.5. Associations of Queens’ Body Size and Body Weight with Production Performance and Biological Traits

**Figure 8:**
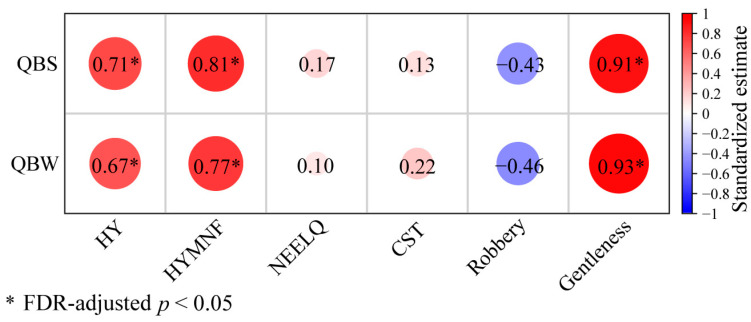
Associations of queens’ body size and body weight with selected colony production performance and biological traits. QBS, queens’ body size; QBW, queens’ body weight; HY, honey yield; HYMNF, honey yield during the main nectar flow; NEELQ, number of effective eggs laid by queen; CST, colony strength. Robbery and gentleness are behavioral traits evaluated in colony performance testing. Circle size and color indicate the magnitude and direction of standardized estimates. Asterisks indicate significant associations after FDR correction (* FDR-adjusted *p* < 0.05).

Although honey bee productivity is expressed at the colony level, the queen is the primary reproductive individual and the main target for genetic evaluation in breeding programs. Therefore, we analyzed the associations of queens’ body size and body weight with selected colony production and biological traits (Figure 8). After FDR correction, both queens’ body size and body weight were significantly positively associated with HY (standardized estimates = 0.71 and 0.67, respectively; FDR-adjusted *p* < 0.05), HYMNF (0.81 and 0.77, respectively; FDR-adjusted *p* < 0.05), and gentleness (0.91 and 0.93, respectively; FDR-adjusted *p* < 0.05). In contrast, the associations with NEELQ (0.17 and 0.10, respectively) and CST (0.13 and 0.22, respectively) were weak and not significant after FDR correction. Robbery showed negative but non-significant associations with queens’ body size and body weight (−0.43 and −0.46, respectively). These results indicate that larger and heavier queens were mainly associated with higher honey production and better colony gentleness, whereas their relationships with egg-laying, colony strength, and robbery should be interpreted cautiously. Based on regional mean values for queen body size and honey-yield traits across the five regions, simple regressions were fitted to provide a quantitative example of the practical breeding implications. Each 1 mm increase in queen body size was associated with an estimated increase of 6.15 kg/colony/year in annual honey yield and 4.00 kg/colony in honey yield during the main nectar flow (Table 2). Although these trends did not reach statistical significance, likely because of the limited number of regions, they provide a tangible estimate of the potential production benefit associated with selecting larger queens.

## 4. Discussion

Honey bees are significant pollinators [33]. *A. cerana* is the native honey bee in China and is widely distributed across the country [34,35]. In 2008, the colonies’ number of *A. cerana* in China was 2.8 million and 5.16 million in 2014 [36]. By the end of 2016, the colonies’ number of *A. cerana* was close to 6 million [37]. This reflects the increasingly important role of *A. cerana* in modern agriculture in China [38]. Consequently, the breeding of *A. cerana* has become more important. Body size and body weight are important traits in honey bee breeding programs [39]. Knowing the correlation between body size and body weight and their effect on productive traits in *A. cerana* are crucial for optimizing breeding objectives.

Body size and body weight are closely related to the health and productivity of honey bee colonies [40,41]. Colonies with large individuals are healthy [40], easy to manage, well productive [42], and could bring great economic benefits. Our study found a significant positive relationship between body size and body weight in *A. cerana*. Importantly, this positive association was further supported by linear mixed-effects models after accounting for colony-level random effects, indicating that the relationship between body size and body weight was not merely driven by non-independence among individuals from the same colony. The coefficient of variation results showed that there was substantial phenotypic variation in body weight of queens and drones. These results indicated that there would be good genetic improvement for selection on emergence body weight in *A. cerana*. Due to the small body size and stinging nature of honey bees, it is not easy to accurately and quickly measure their body size and body weight. This study examined the relationship between body size and body weight in *A. cerana*. The results showed that body size was significantly and positively associated with body weight. Furthermore, a linear mixed-effects model was established to predict body weight from body size while accounting for the hierarchical sampling structure of individuals nested within colonies. This model has practical value for breeding work. In routine manual measurements, body weight is generally easier and faster to obtain because newly emerged bees can be weighed directly in tubes. By contrast, body size measurement requires bees to be photographed in a fully extended posture, which often takes more time because newly emerged bees may move or curl their abdomens. Therefore, direct weighing remains the more convenient method when individual bees can be handled. However, the linear mixed-effects model established in this study indicates that body size can be used to estimate body weight when body-size data are available. This is particularly useful in image-based or automated monitoring systems, where photographs or videos of bees can be collected without weighing each individual. Under such conditions, body size measurements extracted from images may provide an auxiliary basis for estimating body weight and improving the efficiency of morphological evaluation in *A. cerana* breeding.

The analyses of body size and body weight among different bee groups revealed no significant cross-group associations among queens, drones, and workers for either trait. This lack of significant association suggests that body-size and body-weight traits are largely group-specific in *A. cerana*. Although queens, drones, and workers originate from the same colony, their body traits may be influenced by different developmental pathways, nutritional allocation patterns, and biological functions [43]. Therefore, drone and worker measurements may provide useful background information on colony morphology, but they should not be treated as direct proxies for queen body size or body weight in selective breeding. These findings indicate that queen body-type traits should be evaluated directly when they are used as breeding indicators.

The environmental analysis revealed that thermal conditions were the most consistent factors associated with variation in queen and drone body-type traits. Annual minimum temperature and annual mean temperature showed significant negative standardized estimates for queen and drone body size and body weight, suggesting that warmer environments may constrain body-size development and emergence weight accumulation in reproductive bee groups. This pattern is consistent with the Temperature–Size Rule [44], whereby higher temperatures may accelerate developmental rates and increase metabolic costs, leading to smaller adult body size.

Latitude was significantly positively associated with all four body-type traits, indicating a clear geographic gradient in reproductive-group morphology. This result partly supports the expectation that larger body size may be favored in colder or higher-latitude environments because larger individuals have a relatively lower surface-area-to-volume ratio and potentially improved thermal retention [45]. However, elevation and longitude were not significant after FDR correction, and therefore they should be interpreted cautiously rather than as independent environmental drivers in the present dataset.

Annual precipitation showed negative associations with queens’ body size, queens’ body weight, and drones’ body size, but not with drones’ body weight after FDR correction. A possible explanation is that high-precipitation environments may be associated with increased humidity, reduced foraging windows, or altered seasonal resource availability, which could indirectly influence brood-rearing conditions and larval nutritional allocation [40,43]. Because these environmental associations are observational and based on a limited number of regions, they should be interpreted as adaptive patterns or hypotheses rather than evidence of direct causation. Comparative evidence from other honey bee studies supports the view that morphometric traits can vary across ecological and geographic gradients. In *Apis mellifera* (*A. mellifera*), standardized morphometric approaches have long been used to characterize subspecies and ecotypes and to evaluate local adaptation [26]. For example, in West and Central Africa, forewing shape and size in *A. mellifera* differed significantly among ecological zones and were associated with latitude, longitude, and altitude [46]. Similar environmental structuring has also been reported in *A. cerana*. In southwestern China, *A. cerana* populations from high-elevation areas showed larger body sizes and darker coloration than populations from low-elevation basin areas, probably reflecting adaptation to integrated ecological factors such as air temperature, humidity, floral resources, and light intensity [47]. Together with these previous studies, our results suggest that the positive associations with latitude and the negative associations with annual minimum and mean temperatures observed in the present study are consistent with broader honey bee morphometric responses to environmental gradients. However, species-specific genetic backgrounds, local ecological conditions, and colony management practices may modify these relationships; therefore, further comparative studies between *A. cerana* and *A. mellifera* are needed.

The production-trait analysis further supports the practical breeding relevance of queen body-type traits. As shown in Figure 8, both queen body size and queen body weight were significantly positively associated with annual honey yield and honey yield during the main nectar flow, indicating that larger or heavier queens may be linked to colonies with stronger honey-production potential. The strongest associations were observed with gentleness, suggesting that colonies headed by larger or heavier queens tended to show more favorable handling behavior. However, the associations with NEELQ, CST, and robbery were not significant after FDR correction; therefore, these traits should not be used as direct evidence that larger queens necessarily improve egg-laying performance, colony strength, or anti-robbery behavior. Because these analyses are observational, the results should be interpreted as breeding-relevant associations rather than causal effects.

It should be noted that this study has several limitations. First, the relationships between queen morphology and colony productive traits were based on observational correlations and therefore cannot demonstrate causality. Colony performance may also be affected by unmeasured factors, such as management practices, nutrition, disease status, floral resource availability, queen mating quality, and genetic background. Therefore, these results should be interpreted as associations, and further controlled experiments are needed to verify whether selection for queen body size or body weight can directly improve colony productivity in *A. cerana*. Another limitation is that no formal immobilization method, such as CO_2_ anesthesia or cooling, was used during body size imaging. Although newly emerged bees were relatively inactive and were photographed only when the body was naturally extended, slight residual variation in abdominal posture may still have introduced measurement error in straight-line body size estimates. Future studies should consider using standardized immobilization procedures or repeated-image measurements to further improve measurement precision. Also, because this study used a single longitudinal measure of body size, it may not capture all aspects of honey bee morphometric variation. Future studies should incorporate additional stable morphological indicators, such as wing length, thorax width, or head capsule width to provide a more comprehensive evaluation of body size in *A. cerana*.

## 5. Conclusions

Honey bees are important contributors to crop pollination and agricultural production, and they provide substantial economic benefits for beekeepers. Body size and body weight are important breeding traits. This study found a significant positive relationship between body size and body weight in *A. cerana*, and this relationship remained significant after colony-level random effects were accounted for using linear mixed-effects models. In conclusion, body size and body weight were significantly and positively correlated within queens, drones, and workers, indicating that body size can reflect body weight to some extent within the same bee group. However, correlations among different bee groups were weak and non-significant after FDR correction. These results suggest that body-size and body-weight traits are group-specific, and drone or worker traits should not be used as direct substitutes for queen traits in selective breeding. As the reproductive bee groups that transmit genetic material within colonies, queens and drones showed positive associations of body size and body weight with latitude, but negative associations with annual minimum temperature and annual mean temperature after FDR correction. Annual precipitation was also negatively associated with most queen and drone body-type traits. Moreover, queens’ body size and body weight were significantly positively associated with HY, HYMNF, and gentleness after FDR correction, whereas their associations with NEELQ, CST, and robbery were not significant. These findings suggest that queen body-size and body-weight traits may provide useful auxiliary indicators for selecting colonies with higher honey yield and improved gentleness, but they should be combined with direct colony-performance records in breeding practice. Overall, this study provides an important empirical basis for the selective breeding of body-type traits in *A. cerana*, facilitating the optimization of breeding objectives and the acceleration of genetic improvement.

## Figures and Tables

**Table 1:** Linear mixed-effects model results for the relationship between body size and body weight within each bee group of *A. cerana* from Jinfo Mountain.

Bee Group	*n*	Colonies	Fixed Effect	Estimate	SE	*p*-Value	Model Equation
Virgin queens	150	15	Body size	0.1157	0.0099	<0.001	QBW = −35.660 + 0.1157 × QBS
Drones	250	15	Body size	0.0866	0.0146	<0.001	DBW = 14.368 + 0.0866 × DBS
Workers	330	15	Body size	0.0337	0.0043	<0.001	WBW = 37.909 + 0.0337 × WBS

Note: QBS, queens’ body size; QBW, queens’ body weight; DBS, drones’ body size; DBW, drones’ body weight; WBS, workers’ body size; WBW, workers’ body weight. Body size expressed as mm × 100. Linear mixed-effects models were fitted as body weight ~ body size + (1|colony) separately for virgin queens, drones, and workers. Colony identity was included as a random intercept.

**Table 2:** Regression estimates of expected changes in honey yield per 1 mm increase in queen body size based on five-region mean data.

Response Variable	Regression Equation	Expected Change per 1 mm Increase in QBS	R^2^	*p*-Value
HY (kg/colony/year)	HY = −80.41 + 6.15 × QBS	+6.15 kg/colony/year	0.619	0.114
HYMNF (kg/colony)	HYMNF = −53.46 + 4.00 × QBS	+4.00 kg/colony	0.680	0.086

Note: QBS, queen body size; HY, annual honey yield; HYMNF, honey yield during the main nectar flow. Regression analyses were based on five-region mean data. These estimates should be interpreted as associative rather than causal. In this analysis, QBS was expressed in mm.

## Data Availability

The original contributions presented in this study are included in the article material. Further inquiries can be directed to the corresponding author.

## Abbreviations

The following abbreviations are used in this manuscript:

AMAT	Annual maximum temperature
AMIT	Annual minimum temperature
AMET	Annual mean temperature
AP	Annual precipitation
CI	Confidence interval
CST	The number of frames covered by adult workers and brood
DBS	Drones’ body size
DBW	Drones’ body weight
FDR	False discovery rate
HY	The total marketable honey produced per colony per year
HYMNF	The harvestable honey obtained during peak nectar secretion
LMM	Linear mixed-effects model
NEELQ	The mean number of eggs per day that developed into pupae
QBS	Queens’ body size
QBW	Queens’ body weight
SD	Standard deviation
SE	Standard error
WBS	Workers’ body size
WBW	Workers’ body weight
